# Single-Cell Transcriptional Profiling of Mouse Islets Following Short-Term Obesogenic Dietary Intervention

**DOI:** 10.3390/metabo10120513

**Published:** 2020-12-18

**Authors:** Annie R. Piñeros, Hongyu Gao, Wenting Wu, Yunlong Liu, Sarah A. Tersey, Raghavendra G. Mirmira

**Affiliations:** 1 Department of Pediatrics, Indiana University School of Medicine, Indianapolis, IN 46202, USA; apineros@iupui.edu (A.R.P.); wuwent@iu.edu (W.W.); 2 Department of Medical and Molecular Genetics, Indiana University School of Medicine, Indianapolis, IN 46202, USA; hongao@iu.edu (H.G.); yunliu@iu.edu (Y.L.); 3 Kolver Diabetes Center and Department of Medicine, The University of Chicago, Chicago, IL 60637, USA; stersey@uchicago.edu

**Keywords:** islet, obesity, insulin, transcriptomics

## Abstract

Obesity is closely associated with adipose tissue inflammation and insulin resistance. Dysglycemia and type 2 diabetes results when islet β cells fail to maintain appropriate insulin secretion in the face of insulin resistance. To clarify the early transcriptional events leading to β-cell failure in the setting of obesity, we fed male C57BL/6J mice an obesogenic, high-fat diet (60% kcal from fat) or a control diet (10% kcal from fat) for one week, and islets from these mice (from four high-fat- and three control-fed mice) were subjected to single-cell RNA sequencing (sc-RNAseq) analysis. Islet endocrine cell types (α cells, β cells, δ cells, PP cells) and other resident cell types (macrophages, T cells) were annotated by transcript profiles and visualized using Uniform Manifold Approximation and Projection for Dimension Reduction (UMAP) plots. UMAP analysis revealed distinct cell clusters (11 for β cells, 5 for α cells, 3 for δ cells, PP cells, ductal cells, endothelial cells), emphasizing the heterogeneity of cell populations in the islet. Collectively, the clusters containing the majority of β cells showed the fewest gene expression changes, whereas clusters harboring the minority of β cells showed the most changes. We identified that distinct β-cell clusters downregulate genes associated with the endoplasmic reticulum stress response and upregulate genes associated with insulin secretion, whereas others upregulate genes that impair insulin secretion, cell proliferation, and cell survival. Moreover, all β-cell clusters negatively regulate genes associated with immune response activation. Glucagon-producing α cells exhibited patterns similar to β cells but, again, in clusters containing the minority of α cells. Our data indicate that an early transcriptional response in islets to an obesogenic diet reflects an attempt by distinct populations of β cells to augment or impair cellular function and/or reduce inflammatory responses as possible harbingers of ensuing insulin resistance.

## 1. Introduction

An analysis of the National Health and Nutrition Examination Survey database revealed that the crude prevalence of prediabetes and diabetes in the US exceeds 50% [1]. Cardiovascular consequences, including stroke, myocardial infarction, and mortality, increase even as blood glucose rises in the prediabetic phase [2]. Type 2 diabetes (T2D) has been increasing in incidence in the USA in a manner that has tracked closely with the increasing prevalence of abdominal obesity [3]. Notably, across the spectrum from obesity to T2D, insulin resistance appears to be a common feature, and as such, insulin-producing islet β cells are considered central determinants in the transition from normoglycemia to dysglycemia. The gradual or sudden failure to secrete sufficient insulin heralds rising glucose levels [4]. Precisely how and why β cells fail have been the subjects of intense investigation in recent years. One point of view posits that the increased demand for insulin secretion in the setting of insulin resistance overwhelms the capacity of β cells to produce insulin, largely as a result of the incapacity of the endoplasmic reticulum to accommodate protein throughput [5]. Another perspective suggests that excessive circulating proinflammatory cytokines or free fatty acids trigger intracellular signaling cascades that lead to β-cell inflammation, oxidative stress, and possibly cell death [6]. Finally, recent data have suggested the provocative hypothesis that secretory failure and apoptosis may only represent minor components, and the dedifferentiation of β cells to a precursor-like state effectively leads to reduced functional β-cell mass [7]. Regardless of the primary pathology, a better understanding of the molecular processes occurring in the β cell during early obesity and dysglycemia might permit targeted therapeutic interventions that allow for disease modification.

With the advent of single-cell RNA sequencing (sc-RNAseq) technologies [8], important new insights have been emerging about the nature of cells in the pancreatic islet. For example, it was shown that α cells and β cells of the adult human islet exist in multiple subpopulations, and some of those from adults with T2D exhibit features similar to those of children, supporting the dedifferentiation hypothesis [9]. Data of this sort both illuminate the potential to extract information that would be masked using bulk sequencing strategies and emphasize the heterogeneity that exists within the islet endocrine cell pool and how specific populations might have broader implications for emerging metabolic phenotypes in an organism. In light of these and other findings, in this study, we asked if alterations in islet cell gene expression patterns occur in the earliest phases of obesity and dysglycemia in a mouse model of evolving obesity and specifically how these changes might have the ability to portend future functional alterations that give rise to overt T2D. Our data show that early changes in β-cell gene expression reflect an ability of distinct β-cell populations to respond to the metabolic challenges during high-fat-diet (HFD) feeding.

## 2. Results

### 2.1. Identification of Islet Cell Type Clusters by sc-RNAseq

**Figure 1:**
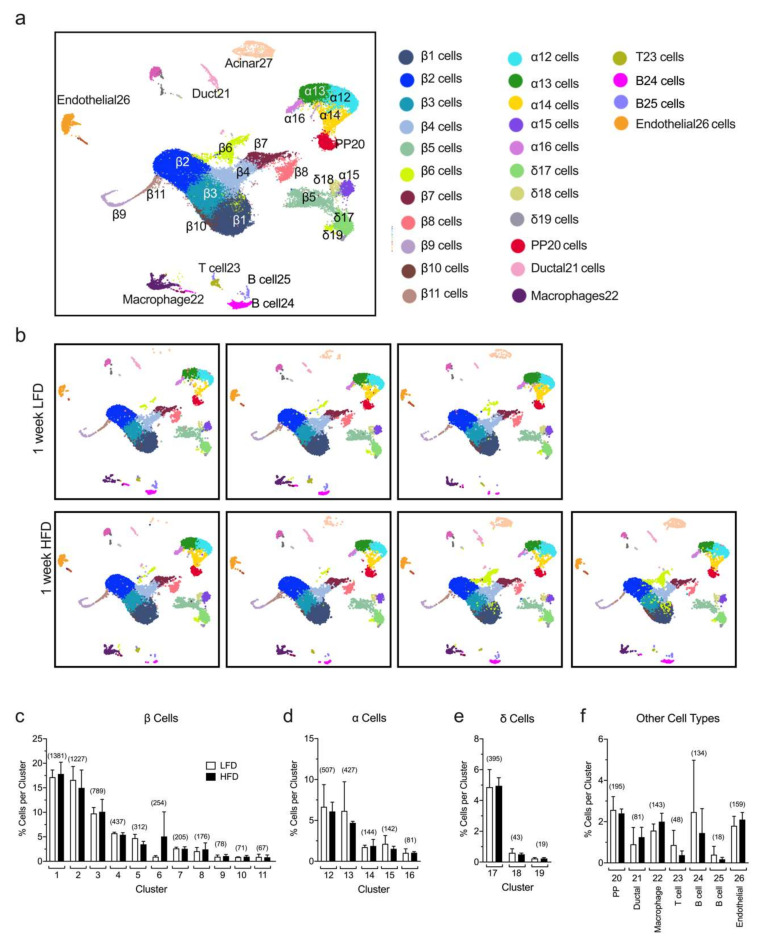
Identification of islet cell type clusters by single-cell RNA sequencing. Single cells were obtained from dissociated islets from male *C57BL/6J* mice fed for one week with either a high-fat diet (HFD, 60% kcal from fat, *n* = 4) or a control low-fat diet (LFD, 10% kcal from fat, *n* = 3) and used to perform single-cell RNA sequencing. (**a**) Annotation of islet cell types into different clusters based on the expression of key identifying genes, depicted in the Uniform Manifold Approximation and Projection for Dimension Reduction (UMAP) plots of merged sc-RNAseq profiles from LFD and HFD mice; (**b**) UMAP of single-cell RNA sequencing profiles from islets of individual mice fed a HFD or a LFD, as indicated; In (**c–f**), the percentage of β cells (**c**), α cells (**d**), δ cells (**e**), and other cell types (**f**) identified per cluster relative to the total number of cells sequenced is shown. Data are mean ± SEM, *n* = 3 for LFD and *n*= 4 for HFD. The number in the *parentheses* above each bar indicates the average number of cells per cluster.

Previous studies from our group demonstrated that a one-week period of HFD (60% kcal from fat) feeding compared to a low-fat diet (LFD, 10% kcal from fat) in mice led to a significant increase in mRNA translation, mTOR pathway activation, and cellular proliferation in pancreatic islets [10,11]. However, these prior studies do not provide the granularity to assess which of the specific endocrine cellular populations account for these responses. To gain more insight into the molecular pathways and the individual changes happening at the single-cell level, we performed sc-RNAseq from a collection of dissociated islets (180–200 islets/mouse) isolated from a total of seven male C57BL/6J mice fed for one week with either a HFD (60% kcal from fat, n = 4) or a control LFD (10% kcal from fat, n = 3). Each mouse sample was processed separately. Our analysis of sc-RNAseq allowed the annotation of islet cell types into different clusters based on the expression of key identifying genes, depicted in the Uniform Manifold Approximation and Projection for Dimension Reduction (UMAP) plots in Figure 1a and Supplementary Figure S1. We identified 26 cell clusters representing 9 distinct cell types based on the gene expression of canonical markers and, as expected, the major populations identified were β cells (Ins1+, Ins2+, Mafa+), α cells (Gcg+, Ttr+, Irx2+), and δ cells (Sst+, Ghsr+, Rbp4+), with smaller populations of other cell types, including PP cells (Ppy+), ductal (Krt19+, Hnf1b+), endothelial cells (Plvap+, Esm1+), immune cells as macrophages (Adgre1+, Lyz2+), T cells (Trbc2+, Cd3g+, Cd4+), and B cells (Cd19+, Igkc+, Ighm+) (Figure 1a). Notably, no differences were observed in the percentage of cells identified in any given cluster in HFD-fed compared to control LFD-fed mice (Figure 1b–f). Furthermore, we found that the percentage of total α, β, and δ cells or other types of cells identified with sc-RNAseq does not change substantially within replicates and between the LFD and HFD groups (Supplementary Table S1). These results demonstrate the reproducibility of our data at a single-cell level.

### 2.2. Single-Cell RNA Sequencing Analysis Reveals Greatest Gene Expression Changes in Minor β Cell Clusters Following Short-Term HFD Feeding

**Figure 2:**
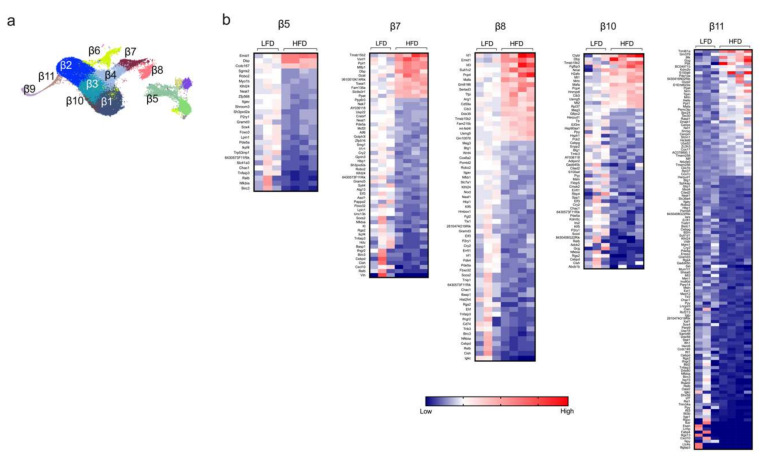
Identification of differentially expressed genes of the minor β-cell clusters. Β-cell clusters were identified from dissociated islets from male *C57BL/6J* mice fed for one week with either a high-fat diet (HFD, 60% kcal from fat) or a control low-fat diet (LFD, 10% kcal from fat). (**a**) Representative UMAP plot of β-cell clusters identified by single-cell RNA sequencing; (**b**) heatmaps of the minor β-cell clusters of genes significantly differentially expressed (*p* < 0.05) in the β-cell clusters β5, β7, β8, β10, and β11; genes are ordered from most positive to most negative fold-change.

Next, we examined the gene expression profiles in β cells from mice fed either a HFD or LFD for one week. UMAP analysis identified a total of 11 distinct β-cell clusters (β1-β11, see Figure 1a and Figure 2a). Based on the proportion of cells per cluster (Figure 1c), we identified three major clusters of β cells (β1–β3) and eight minor clusters (β4–β11). Differential gene expression between HFD and LFD were interrogated, and statistical significance was determined by using edgeR on the integrated single-cell data obtained by the R package Seurat (see Section 4). It is notable that the major clusters of β cells (β1–β3) showed minimal change in gene expression patterns (Supplementary Figure S2), whereas the greatest changes were observed in the minor clusters (most notably β5, β7, β8, β10, β11) (Figure 2b). These findings suggest that minor β-cell clusters drive the earliest responses to HFD, and further emphasize how bulk RNA sequencing might miss these sentinel changes. In these minor clusters, the most notable gene expression changes reflect upon hormone secretion and intracellular inflammatory pathways. Clusters β5, β7, β8, β10, and β11 demonstrated significant *decreases* in genes that promote insulin secretion (*Atf6, Meg3, Herpud1*), β cell survival (*Sox4, Tnfaip3*), calcium signaling (*Robo2*), and activation of immune response (*Nfkb1, Il1r1, Cxcl10, Ifngr2*) (Figure 2b), with increases in genes that block insulin release (*Mt2*) and promote apoptosis (*Mif*) [12,13,14,15,16,17]. Furthermore, we observed an increased expression of *Mafa*, a gene that encodes a transactivator of the *Ins1/2* genes, in this group of clusters following HFD feeding.

Among the β-cell clusters that showed minimal gene expression changes between HFD and LFD, four of them (β1, β2, β6, and β9) showed downregulation of genes for the endoplasmic reticulum stress response (*Chac1*) and activation (*Nfkbia, Cxcl10*) of inflammatory processes (Supplementary Figure S2) [18,19]. Clusters β3 and β4 showed an increase in expression of genes associated with insulin secretion (*Dbp*) and a complementary decrease in expression of genes encoding a suppressor of insulin signaling (*Pdea5*) and inflammatory pathways (*Nfkbia, Tnfaip3*) (Supplementary Figure S2) [20,21].

Collectively, these gene expression changes provide a picture of the response of β cells to HFD feeding—namely, that specific, larger clusters of β cells demonstrate augmented insulin secretory capacity and reduction of ER stress, whereas smaller clusters exhibit a reduced insulin secretion capacity and survival. It remains unclear if these findings signify an early response of small clusters, which will be later reflected in larger clusters as the impact of HFD is prolonged, or if they reflect what will be a persistent, and perhaps competing, heterogenous response among β-cell subtypes whose function on-balance determines T2D outcomes. In this regard, our prior studies of HFD feeding in male mice [22] were suggestive of recurring patterns of β-cell loss, wherein it was suggested that different subpopulations of β cells might have shown evolving susceptibility to death or dedifferentiation as HFD feeding progressed.

Using the same diet that we have used in the present work, other studies from our group have demonstrated that although one week of HFD feeding results in only minor impairments of glucose tolerance, longer-term HFD feeding impairs both glucose tolerance and insulin secretion [10,11,22]. Therefore, further studies would be necessary to know if β-cell dysfunction in the smaller clusters drives hyperglycemia and impairs insulin secretion during obesity. Several studies have also demonstrated that proinflammatory signals, cellular stress, and genetic components contribute to T2D development [23,24,25,26]. Here, we found that despite the HFD feeding, β-cell clusters overall tend to decrease the expression of proinflammatory genes, suggesting that cellular stress and β-cell dysfunction are the initial triggers that propagate the inflammatory response in the HFD feeding model. However, more studies will be necessary to clarify if the events of cellular stress and β-cell dysfunction identified in the smaller clusters of β cells propagate cellular stress in other larger clusters. 

### 2.3. Gene Pathway Analysis Reveals Molecular Responses Related to Inflammation/Immunity and Oxidative Stress in β Cells Following HFD Feeding

**Figure 3:**
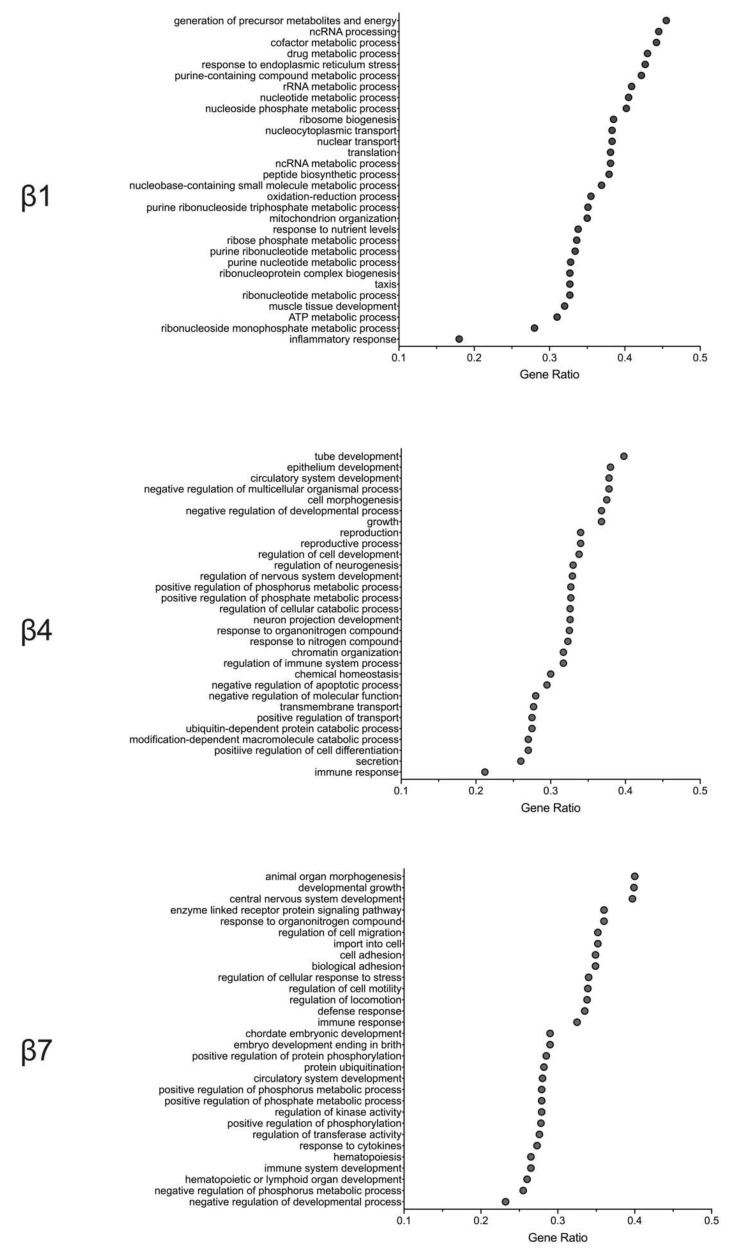
Altered signaling pathways of the minor β-cell clusters. Gene ontology gene set enrichment analysis (GO GSEA) was performed after single-cell RNA sequencing to evaluate the signaling pathways specifically altered by high-fat-diet feeding vs. low-fat-diet feeding in different β-cell clusters. Shown are the GO GSEA analyses of β1, β4, and β7 cell clusters.

Whereas the preceding studies focused on specific islet-cell gene expression changes between HFD- and LFD-fed mice, they do not provide an unbiased context for how these alterations affect functional signaling pathways in the cell. Therefore, we performed gene ontology gene set enrichment analysis (GO GSEA) to evaluate the signaling pathways specifically altered by HFD feeding in different β-cell clusters. We identified three general pathway responses exemplified by clusters β1, β4, and β7 (Figure 3). β-cell clusters with minimal gene expression changes in response to HFD had an overrepresentation of gene pathways in the response to cellular metabolism, endoplasmic reticulum (ER) stress, and oxidation–reduction process (Figure 3). A second pattern, seen largely among clusters β3 and β4 (Figure 3), showed an overrepresentation of genes associated with cell differentiation/development, immune response, and the negative regulation of apoptosis. Finally, a third group, exemplified by cluster β7 and exhibited the greatest gene expression changes in response to a HFD, showed changes in gene pathways that modulate immune/inflammatory response and cellular stress (Figure 3). Collectively, these pathway analyses are suggestive of β-cell adaptations to HFD feeding in which inflammation/immune cascades are affected and in which changes to cellular redox and metabolite utilization prevail. Our findings are consistent with the observations in the literature that free fatty acids present in HFDs impose oxidative stress [6] and enhance signaling pathways linked to cytokines and inflammation [27].

#### Short-Term HFD Feeding Reveals Heterogeneity in α Cell Responses

**Figure 4:**
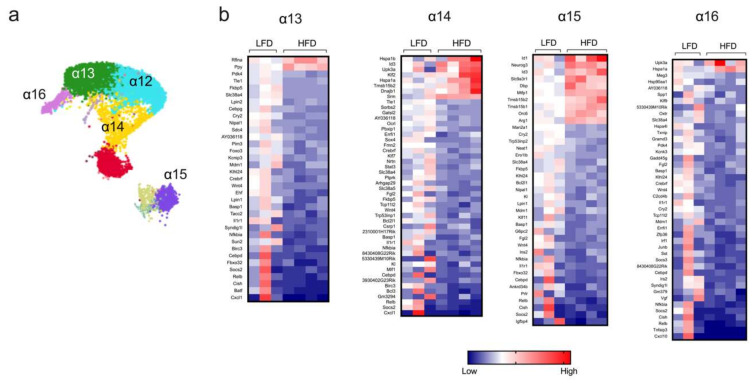
Transcriptome profile in α-cell clusters identified by single-cell RNA sequencing. α-cell clusters were identified following single-cell RNA sequencing from male *C57BL/6J* mice fed for one week with either a high-fat diet (HFD, 60% kcal from fat) or a control low-fat diet (LFD, 10% kcal from fat). (**a**) Representative UMAP plot of α-cell clusters; (**b**) heatmaps depicting genes differentially regulated (*p* < 0.05) in α-cell clusters; genes are ordered from most positive to most negative fold-change.

Islet α cells produce glucagon, a major insulin counterregulatory hormone. The dysregulated hypersecretion of glucagon contributes to dysglycemia in obesity and T2D [28]. Bulk RNA-seq studies from long-term HFD-fed mice previously demonstrated that α cells display remarkably minor changes in transcriptome profile [29], but such studies might belie greater changes in gene expression patterns in specific subsets of α cells. In this study, sc-RNAseq identified minor distinct clusters of α cells (designated α12–α16, Figure 4a), the populations of which were not altered upon HFD feeding of mice for one week (Figure 1d). Differential gene expression analysis of these clusters was then performed. The cluster containing the largest pool of α cells (α12) did not show statistically significant changes in gene expression; however, clusters α13, α14, α15, and α16 showed multiple differentially expressed genes in HFD-fed mice compared to LFD-fed mice (Figure 4b). Notably, we observed that HFD feeding led to a decrease in genes associated with inflammation (*Il1r, Cxcl1*, *and Nfkbia*) in all four α-cell clusters (Figure 4b). Only cluster α13 showed changes in hormone expression, with an increase in Ppy after HFD feeding, indicating that, in general, short-term HFD does not lead to the misexpression of hormone-encoding genes. Other general observations include an increase in the expression of genes involved in cell survival and proliferation (*Upk3a*) and cellular stress (*Hspa1a* and *Hspa1b*) in clusters α14 and α16 [30,31], the enhanced expression of genes related to endocrine progenitors (*Neurog3*), and an oxidative stress/ER stress (*Ero1b*) decrease in cluster α15 [32,33]. Collectively, these data indicate that, like β cells, the early gene expression changes in α cells (a) are dependent on the specific cluster and thereby exhibit cluster-dependent heterogeneity, and (b) might be missed by bulk sequencing approaches since a major cluster of α cells exhibits no significant changes.

## 3. Discussion

To our knowledge, no studies to date have assessed the heterogeneity of individual islet cell responses to HFD feeding in mice. The use of sc-RNAseq technologies allows us to employ clustering analysis to segregate populations of cells that exhibit similar gene expression patterns and identify the heterogeneity of cellular responses. In this study, we were interested in the early responses of islet endocrine cells to HFD feeding. Prior studies have shown that one week of 60% kcal from fat a HFD results in mild glucose intolerance in male C57BL/6 mice [10,34], suggesting that β-cell failure in the setting of HFD-induced insulin resistance may occur early during obesity. Our results from sc-RNAseq analysis show that the minor clusters of β cells (β5, β7, β8, β10, and β11) exhibit an impairment in β cell function and survival, whereas some of the major clusters of β cells (β3 and β4) demonstrate transcriptomic changes consistent with a compensatory response; however, others (β1 and β2) still exhibit no statistically significant change. Our findings, therefore, suggest that the failure of a majority of β cells to compensate for prevailing insulin needs might portend the eventual development of hyperglycemia/T2D. Further studies that include analysis of several time points would provide more insight into whether the defective early response found in the minor β cells clusters triggers T2D or if it is just the result of a compensatory response to an obesogenic diet. Similarly, the robustness of a large proportion of α cells, which exhibit gene expression changes that promote proliferation and survival, might permit the more robust production of glucagon that exacerbates dysglycemia. Our studies support the concept that the balance of different populations of cell types in the islet (those more capable of responding to prevailing stress vs. those less capable) might account for the eventual risk for the development of dysglycemia/T2D in the setting of HFDs. 

Several limitations should be noted in our study. First, our study was limited to an early period (one week) following obesogenic diet exposure. Therefore, we cannot know if/how gene expression responses change with time. Do the responsive β-cell clusters remain robustly responsive in their gene expression patterns with more prolonged feeding? Do the less responsive clusters become more responsive with time? Are specific clusters more susceptible to apoptosis or dedifferentiation over time? Does the islet isolation process influence mRNA expression? In future studies, applying our annotation and clustering analyses should allow us to address these questions using timed feeding cohorts. Second, our study was limited to male C57BL/6J mice, which are known to be the most susceptible to the deleterious effects of HFD feeding. The use of mice with more robust islet responsiveness, such as the C57BLKs/J strain [35] or female C57BL/6J mice [36], is likely to provide more insight into the nature and number of β-cell clusters that are responsive to the demand for insulin production. Third, the relevance of our study to human obesity and T2D remains unclear because of species differences and the added genetic heterogeneity in humans that compounds the inherent heterogeneity of the islet cell types. Fourth, although our data are compared to controls, because the procedure of islet isolation is a stressful process, we cannot rule out that it could lead to more exaggerated changes in gene expression profile that may impact our findings. Finally, we should note that sc-RNAseq may underestimate the nature of gene expression changes since the depth of sequencing by single-cell technologies is considerably lower than that seen in bulk sequencing technologies. Thus, our findings regarding the minimal gene expression changes seen in the majority of β-cell clusters may not fully report actual changes occurring in these cells. Nevertheless, our studies provide new biologic insight into the early changes in gene expression that occurs in islet cell subsets in the early phase of obesogenic diets.

## 4. Materials and Methods

### 4.1. Animal Studies

Male C57BL/6J mice were purchased from Jackson Laboratories at 7 weeks of age. All mice were kept on a standard 12 h–12 h light–dark cycle with ad libitum access to chow and water. Mice were maintained at the Indiana University vivarium according to protocols approved by the Institutional Animal Care and Use Committee. At 8 weeks of age, animals were fed either a LFD (10% kcal from fat; Research Diets D12450B) or a HFD (60% kcal from fat; Research Diets D12492) for one week. 

### 4.2. Islet Isolation

Mouse islets were isolated from collagenase-perfused pancreata, as previously described [37]. After isolation, mouse islets (approximately 180–200 islets) were handpicked and digested with Accutase (EMD Millipore Corporation, Temecula, CA, USA) containing 2 U/mL of DNAse for 5 min at 37 °C sob agitation (1000 rpm). Digested cell islets were washed several times with PBS+2%FBS to eliminate DNAse and then filtered using a cell strainer (40 μm). Single-cell suspensions and samples with more than 90% viability were used for sc-RNAseq.

### 4.3. Preparation of Single Cell 3′ RNA-seq Library

Single-cell 3′ RNA-seq experiments were conducted using the Chromium single-cell system (10 x Genomics, Inc., Pleasanton, CA, USA) and Illumina sequencers at the Center for Medical Genetics (CMG) of the Indiana University School of Medicine. Each cell suspension, a collection of 180–200 islets isolated from an individual mouse, was first inspected under a microscope for cell number, cell viability, and cell size. For the initial cell suspension with low viability, the single-cell preparation was further processed, which included centrifugation, resuspension, and filtration to remove cell debris, dead cells, and cell aggregates. Single-cell capture and library preparation were performed according to the Chromium Single Cell 3′ Reagent Kits V3 User Guide (10x Genomics PN-1000075, PN-1000073, PN-120262). An appropriate number of cells were loaded on a multiple-channel microfluidics chip of the Chromium Single Cell Instrument (10x Genomics) with a targeted cell recovery of 8000 to 10,000. Single-cell gel beads in an emulsion containing barcoded oligonucleotides and reverse-transcriptase reagents were generated with the V3 single-cell reagent kit (10x Genomics). Following cell capture and cell lysis, cDNA was synthesized and amplified. Illumina sequencing libraries were then prepared with the amplified cDNA. The resulting libraries were assessed with Agilent TapeStation. The final libraries were sequenced using a custom program on Illumina NovaSeq 6000. About 550 million read pairs were generated for each sample, with 28 bp of cell barcode and unique molecular identifier reads and 91 bp RNA reads.

### 4.4. Analysis of sc-RNAseq Sequence Data

CellRanger 3.0.2 (http://support.10xgenomics.com/) was utilized to process the raw sequence data generated. Briefly, CellRanger used bcl2fastq (https://support.illumina.com/) to demultiplex raw base sequence calls generated from the sequencer into sample-specific FASTQ files. The FASTQ files were then aligned to the mouse reference genome mm10 with RNA-seq aligner STAR. The aligned reads were traced back to individual cells, and the gene expression level of individual genes was quantified based on the number of UMIs detected in each cell.

The filtered gene–cell barcode matrices generated from CellRanger were used for further analysis with the R package Seurat (Seurat development version 3.0.0.9200) [38,39] with Rstudio version 1.1.453 and R version 3.5.1. Quality control (QC) of the data was implemented as the first step in our analysis. We filtered out genes that were detected in less than five cells and cells with less than 200 genes. To further exclude low-quality cells in downstream analysis, we used the Outlier function from the R package scatter [40] together with a visual inspection of the distributions of the number of genes, UMIs, and mitochondrial gene content. Cells with an extremely high or low number of detected genes/UMIs were excluded. In addition, cells with a high percentage of mitochondrial reads were also filtered out. After removing likely multiplets and low-quality cells, the gene expression levels for each cell were normalized with the NormalizeData function in Seurat. Highly variable genes were subsequently identified.

To integrate the single-cell data of the HFD and LFD samples, functions FindIntegrationAnchors and IntegrateData from Seurat were applied. The integrated data were scaled, and PCA was performed. Clusters were identified with the Seurat functions FindNeighbors and FindClusters. The FindConservedMarkers function was subsequently used to identify cell cluster marker genes. Cell cluster identities were manually defined with the cluster-specific marker genes or known marker genes. The cell clusters were visualized using the t-Distributed Stochastic Neighbor Embedding (t-SNE) plots and Uniform Manifold Approximation and Projection (UMAP) plots.

To investigate cell cluster/type-specific responses triggered by different diet treatments, a pseudobulk count approach was applied. The counts of each gene of all cells within a cell cluster for each biological replicate were aggregated. In this way, the gene–cell count matrix was transformed into a gene–sample level count matrix. The R package muscat (version 1.0.1, [41]) was employed for generating pseudobulk counts and differential gene expression analysis between conditions with the same cluster. In more details, the Seurat object generated from the integrative analysis was first converted into a SingleCellExperiment object using the as.SingleCellExperiment function in Seurat. The SingleCellExperiment object was then used as input for muscat. Gene–cell count data were aggregated with the function aggregateData, and differential gene expression analysis of the pseudocounts was performed with edgeR [42]. GO gene set enrichment analysis was performed using the R package clusterProfiler [43]. The datasets generated and analyzed in the present study are available in the GEO repository (accession number: GSE162512). 

## Supplementary Materials

The following are available online at https://www.mdpi.com/2218-1989/10/12/513/s1, Table S1: Percentage of cells per cluster identified by sc-RNAseq, Figure S1: Genes used for identification of cell clusters, Figure S2: Identification of differentially expressed genes of the major β cell clusters.
