# Supplementary material for: Single-Cell Transcriptional Profiling of Mouse Islets Following Short-Term Obesogenic Dietary Intervention
Source: Metabolites. 2020 Dec 18;10(12):513. doi: 10.3390/metabo10120513 (PMC7765825; doi:10.3390/metabo10120513)
Supplement: Supplementary file 1 [file metabolites-10-00513-s001.pdf]

Supplemental Table S1. Percentage of cells per cluster identified by sc-RNAseq.

	LFD1	LFD2	LFD3	HFD1	HFD2	HFD3	HFD4
**β cells**	69	66	55	67	68	64	58
**α cells**	12	14	28	16	14	15	16
**δ cells**	4	7	6	6	6	6	5
**Acinar**	0	1	2	0	1	4	8
**Other**	14	11	7	9	10	10	11
**Unclassified**	2	2	2	3	2	2	2
**Total**	100	100	100	100	100	100	100

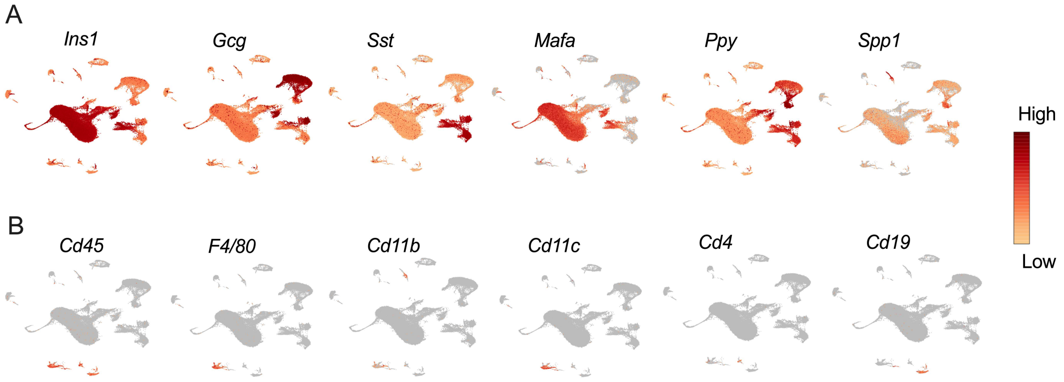

Supplemental Figure S1. Genes used for identification of cell clusters. Single cells were obtained from dissociated islets from male *C57BL/6J* mice fed for one week with either a high fat diet (HFD, 60% kcal from fat) or a control low fat diet (LFD, 10% kcal from fat) and used to perform single-cell RNA sequencing analysis. (**A**) UMAP plots showing spatial expression of identifying genes for the 6 major types of endocrine cells: *Ins1, Gcg, Sst, Mafa, Ppy, Spp1;* (**B**) Expression markers for 5 major populations of immune cells: *Cd45, F4/80, Cd11b, Cd11c, Cd4* and *Cd19*. Color assignments represent levels of expression.

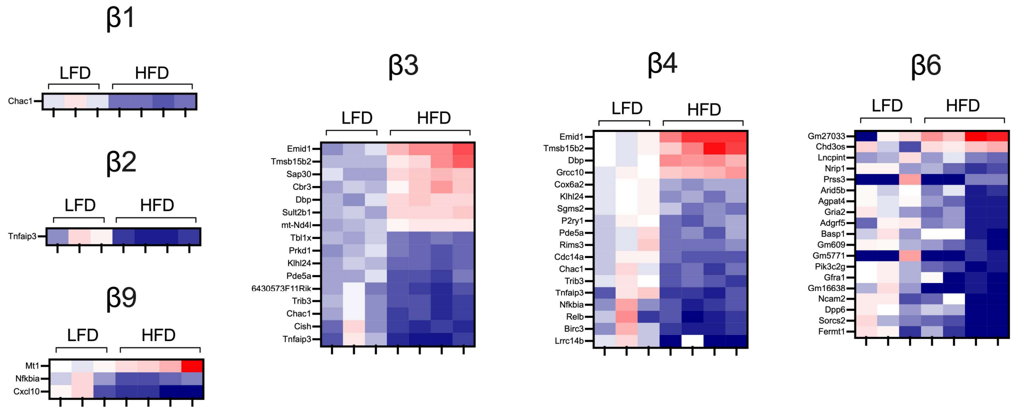

Supplemental Figure S2. Identification of differentially expressed genes of the major β cell clusters. β-cell clusters were identified from dissociated islets from male *C57BL/6J* mice fed for one week with either a high fat diet (HFD, 60% kcal from fat) or a control low fat diet (LFD, 10% kcal from fat). Shown are heatmaps of the major β-cell clusters of genes significantly differentially expressed ($p < 0.05$) in the β-cell clusters; genes are ordered from most positive to most negative fold-change.
